# The ORIN—open reduction and intramedullary nailing—technique for 4-part proximal humerus fractures

**DOI:** 10.1016/j.xrrt.2026.100746

**Published:** 2026-04-11

**Authors:** Edoardo Giovannetti de Sanctis, Bastien Bige, Nicolas Bronsard, Jean-François Gonzalez, Marc-Olivier Gauci

**Affiliations:** IULS- Institut Universitaire Locomoteur et Sports, Pasteur 2 Hospital, CHU, Nice, France

**Keywords:** Proximal humerus fracture, Intramedullary nailing, IMN, PHF, Neviaser, Nail

Four-part proximal humerus fractures (PHFs) continue to present a challenging and controversial fracture pattern to manage due to a high incidence of associated complications.

Indications for a surgical treatment are severely compromised humeral head, nonimpacted fractures with fragment instability, and severely displaced greater tuberosity (GT) and lesser tuberosity (LT).^18^

Locking plate (LP), antegrade intramedullary nailing (IMN), and reverse shoulder arthroplasty are the most commonly used surgical devices to treat 4-part PHFs.^22^ Continued debate exists as to which of these represents the treatment gold standard.

Fixation should be avoided in case of splitting or comminuted displaced humeral head fractures and if associated with moderate to high-grade glenohumeral osteoarthritis.

The goal of any fracture internal device is to achieve a stable fixation, minimizing soft tissue injury.^7^ Concerns exist around the use of LP fixation due to hardware complications: having humeral head-oriented screws increases the risk of humeral head avascular necrosis (AVN), intraarticular screw penetration with glenoid cartilage damages, and tuberosities nonunion and migration. Furthermore, plate screws are parallel to the fracture line, contrasting the current clinical principles of fracture management.

The role of IMN fixation for the treatment of 4-part PHFs has recently regained popularity. Most modern designs (third-generation nails) include short straight nails with proximal divergent and tuberosities-oriented self-locking screws, helping to capture the GT and LT fracture segments, decrease the risk of loss of fixation, and avoid glenoid erosion in case of humeral head necrosis.^9^

The surgical approach used at our institution with the PHF intramedullary nailing varies based on the fracture pattern and fragment displacement.

A percutaneous approach through the Neviaser portal is chosen in case of 2- or 3-part fractures with minimally displaced tuberosities PHF.^9^^,^^10^

However, in case of displaced GT and LT PHFs, the primary goal is to reach an anatomic consolidation of the fragments. The quality of reduction has also been shown to influence the risk of head AVN.^12^^,^^14^ Therefore, 4-part PHFs should not be treated surgically through only a percutaneous approach. In these cases, an open reduction (OR) of the fragments is needed, whereas the intramedullary nail (IN) is still used and inserted through the Neviaser approach.

The purpose of this report is to present our institution’s surgical technique of OR and intramedullary nailing (ORIN) fixation of 4-part PHFs.

## Surgical technique

### Indications and pre-operative planning

**Figure 1:**
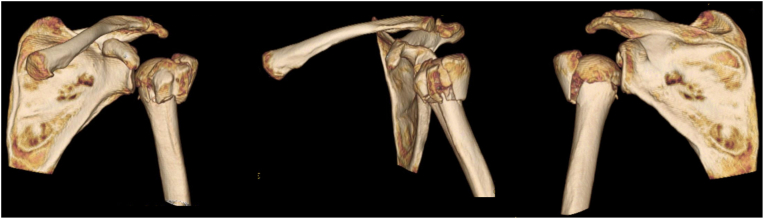
Pre-operative computed tomography scan of a 4-part proximal humerus fracture.

Pre-operatively, the fracture pattern and fragments displacement are assessed with standard radiographs (both a Grashey view and lateral Y view) and a three-dimensional computed tomography scan (Fig. 1).

#### Step 1. Patient positioning

**Figure 2:**
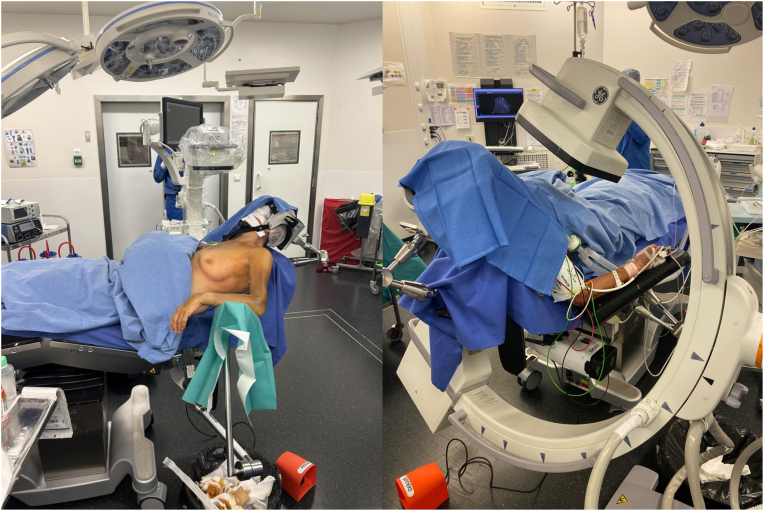
Patients, under general anesthesia added to an interscalene block, are placed in a lazy beach chair position. The C-arm is positioned from the contralateral side of the operative table.

Patients, under general anesthesia added to an interscalene block, are placed in a lazy beach chair position (Table I) (Fig. 2). An adjustable head support is used to fix the patient’s head position, allowing easy access to the shoulder. The operative arm is placed in a pneumatic fully articulating arm support device (Spider Limb Positioner, Smith & Nephew, Andover, MA).

**Table I:** Step-by-step details of the technique.

Step 1. Patient positioning
The patient, under general anesthesia added to an interscalene block, is placed in a lazy beach chair position, with the C-arm positioned from the contralateral side of the operative table.
The anatomic landmarks with the planned incision are marked.
Step 2. The superior approach
The superior (or saber-cut) approach is performed.
The raphe between the anterior and middle head of the deltoid is identified and split.
An anterolateral acromion osteotomy is performed using a straight osteotome.
The fracture site is cleared from hemorrhagic bursa, debris, and blood clots.
Step 3. Fragments reduction (OR – open reduction)
The GT and LT are mobilized to the head segment with the Greater Tuberosity Clamps
The biceps tendon tenodesis is performed within the groove.
Four double-stranded loop sutures (NICELOOP; Tornier, Inc., Bloomington, MN, USA) of different colors are placed through the subscapularis and the external rotators.
The humeral head is derotated and reduced; the tuberosity fracture segments are fixed temporarily with a pointed reduction clamp.
Step 4. Intramedullary nailing (IN)
The nail entry point is reached through an anterolateral or Neviaser approach.
The cannulated antegrade nail—Tornier Humeral Nail (Kalamazoo, MI) is advanced through an external jig with the arm in neutral rotation
The pointed reduction clamp is removed from the tuberosities.
The 4 nonabsorbable double-stranded horizontal rotator cuff loop sutures are passed either medial or lateral to the stem and through the contralateral tendon.
The loop sutures are tightened with the arm in neutral rotation.
Three proximal locking screws are placed through the external targeting device to fix the tuberosities to the head segment.
The 2 divergent distal screws are placed in the diaphysis and fluoroscopically verified
Step 5. Closure
Close the interval between the anterior and middle head of the deltoid
Close in layers the subcutaneous plane and then the skin with Steri-Strips 3M.

*GT*, greater tuberosity; *LT*, lesser tuberosity.

An adequate fluoroscopic visualization of the fracture and humeral shaft should be confirmed before draping. The C-arm is positioned from the contralateral side of the operative table (Fig. 2). Two fluoroscopic images are used: the Grashey view and the lateral Y view.

**Figure 3:**
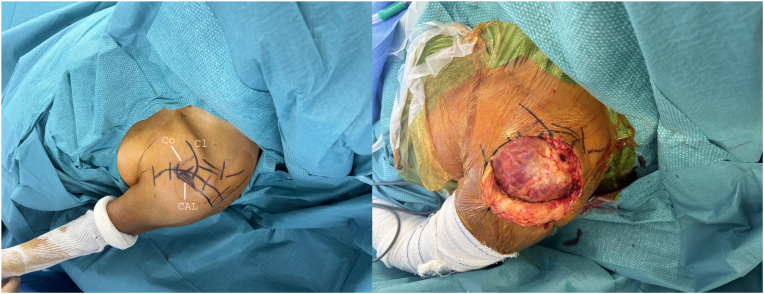
Important surface landmarks are marked, such as the coracoid, acromion, and the approach planned incision: the superior (or saber-cut) approach. *Co*, coracoid; *CAL*, coracoacromial Ligament; *Cl*, clavicle.

The arm is then disinfected with a povidone-iodine solution, and sterile drapes are applied to isolate the shoulder. Important surface landmarks are marked, such as the coracoid, acromion, and the approach planned incision: the superior (or saber-cut) approach (Fig. 3). An iodine-impregnated adhesive plastic incisional drape is then applied.

#### Step 2. The superior approach

A 10 cm straight incision centered 1 cm medial to the lateral border of the acromion, extending from the spine (1 cm medial to the posterolateral corner of the acromion) to the front, over the humeral head in the deltopectoral space.

Two subcutaneous flaps are created laterally and medially to ensure adequate visualization of the deltotrapezial fascia. The lateral flap is retracted, and the wound is kept open using 2 No.1 polydioxanone traction sutures to provide adequate visualization.

The raphe between the anterior and middle head of the deltoid, starting normally from the anterolateral corner of the acromion, is identified and split in the line of the fibers distally for approximately 4 cm from the lateral edge of the acromion to avoid any injury to the axillary nerve. Once the split has been performed, the heads of the deltoid are held back by a Gelpi retractor.

**Figure 4:**
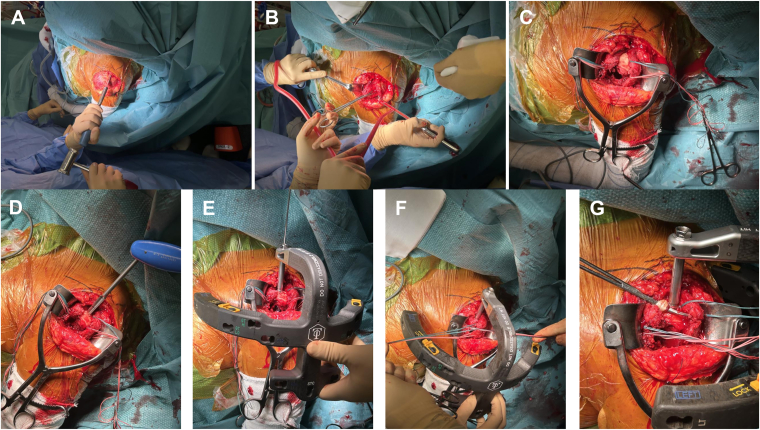
An anterolateral acromion osteotomy is performed using a straight osteotome (**A**). The GT and LT are mobilized to the head segment with the Greater Tuberosity Clamps (**B**). Four double-stranded loop sutures (NICELOOP; Tornier, Inc., Bloomington, MN, USA) of different colors are placed through the subscapularis and the external rotators for GT and LT fixation (**C**). The nail entry point might be reached through the same incision as shown in this case, passing laterally to the acromion (**D**). The cannulated antegrade nail—Tornier Humeral Nail (Kalamazoo, MI, USA) is advanced through an external jig with the arm in neutral rotation, with the aim of achieving adequate reduction of the humeral diaphysis in rotation (**E**). The pointed reduction clamp is removed from the tuberosities (**F**). The intra-articular biceps tendon is removed, once fixed distally within the groove (**G**). *GT*, greater tuberosity; *LT*, lesser tuberosity.

A partial osteotomy of 0.5 mm of thickness of the anterolateral tip of the acromion is performed using a straight osteotome (Fig. 4*A*). The anterolateral corner of the acromion is identified. The osteotome is first oriented posteriorly and then is turned anteriorly to harvest a piece of bone of approximately 5 mm still attached to the anterior head of the deltoid.

#### Step 3. Fragments reduction (OR–open reduction)

The fracture site is cleared from hemorrhagic bursa, debris, and blood clots to facilitate visualization of the rotator cuff tendons (GT and LT), allowing for subsequent fracture reduction. The status of the rotator cuff is inspected.

The biceps tendon tenodesis is performed at this stage with the most proximal portion of the pectoralis major tendon using a No 1 Vicryl (Ethicon, Inc.) with the elbow in full extension. The Gelpi retractor is then replaced with a Kolbel-type self-retaining retractor.

If the GT and LT are still in continuity, the vertical fracture separating partially the 2 fragments is identified and is completed by incising the supraspinatus tendon in line with its fibers.

Otherwise, the tuberosity displacement is identified. The GT and LT are mobilized to the head segment with the Greater Tuberosity Clamps (Fig. 4*B*). Four double-stranded loop sutures (NICELOOP; Tornier, Inc., Bloomington, MN, USA) are applied for GT and LT fixation. Two sutures of different colors are placed through the external rotator cuff tendons: one in the infraspinatus and one in the teres minor. Two sutures of different colors are placed through the subscapularis: one through the upper round tendon previously individualized and one through the middle flat tendon (Fig. 4*C*).

Then, optionally 2 holes at the same level are drilled in the lateral cortex of the diaphysis. Two sutures, in the orthogonal direction, are placed through these cortex holes, with the goal of applying 4 horizontal and 2 vertical cerclages.

The displaced humeral head might then be derotated and reduced if needed with either Steinmann pins or a Kirschner wire. The humeral head stabilization is reached only after tuberosities reduction and fixation. The tuberosity fracture segments are fixed temporarily with a pointed reduction clamp. The position of the head complex is then checked visually and under fluoroscopy.

#### Step 4. Intramedullary nailing (IMN)

The nail entry point might be reached through the same incision as shown in this case, passing laterally to the acromion (Fig. 4*D*). Differently, when the acromial anatomy does not allow reaching the correct entry point, the Neviaser portal might be used to insert the nail percutaneously.

The nail entry point is identified; then the cannulated starter bone is inserted through the supraspinatus muscle and at the vertex of the humeral head through the cartilage (1 cm from the footprint). In case of use of the Neviaser portal, a fluoroscopic control is achieved.

A guide wire is introduced through the bone within the medullary canal. A 9-mm reamer is then inserted over the guide wire and used to create the start cortical hole. The cannulated antegrade nail—Tornier Humeral Nail (Kalamazoo, MI) is advanced through an external jig with the arm in neutral rotation, with the aim of achieving adequate reduction of the humeral diaphysis in rotation (Fig. 4*E*). Indeed, the diaphysis is displaced in adduction and internal rotation due to the pull of 3 internal rotators (pectoralis major, latissimus dorsi, and teres major).^18^

The cannulated antegrade nail might also be used a joystick to further reduce the humeral head.

The pointed reduction clamp is removed from the tuberosities (4.F).

**Figure 5:**
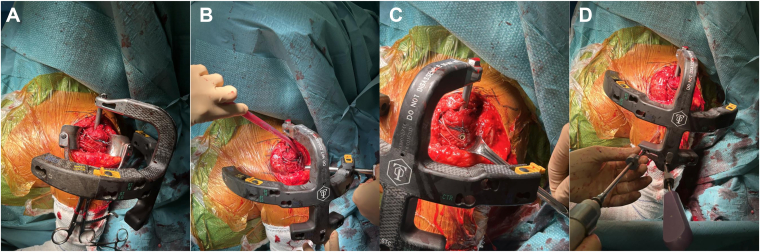
The loop sutures are tightened with the arm in neutral rotation. (**A**). The proximal locking screws placed through the external targeting device fix the tuberosities to the head segment (**B** and **C**). The 2 divergent distal screws are placed in the diaphysis and fluoroscopically verified (**D**).

The 4 nonabsorbable double-stranded horizontal rotator cuff loop sutures (NICELOOP; Tornier, Inc., Bloomington, MN, USA) with different colors are passed either medial or lateral to the stem and through the contralateral tendon to fix the greater with the lesser tuberosity. The 2 optional diaphysis loop sutures (one for each tuberosity) are then passed through, respectively, the external and internal rotator cuff. A pointed reduction clamp is placed again to fix temporarily the tuberosity fracture segments. The sutures are then tightened with the arm in neutral rotation (Fig. 5*A*).

The adequate rotation of the nail is determined: the proximal locking screws placed through the external targeting device should fix the tuberosities to the head segment (Fig. 5*B*). Three (2 for the GT and 1 for the LT) proximal locking screws are inserted, whether percutaneously or using the previously performed lateral approach (Fig. 5*C*). The proximal locking screw length should be fluoroscopically verified. We normally use 36 and 32 mm, respectively, for males and females. The interval between the anterior and posterosuperior cuff is closed with absorbable No. 1 Vicryl (Ethicon, Inc.). Finally, the 2 divergent distal screws are placed in the diaphysis and fluoroscopically verified (size 24 mm or 26 mm) (Fig. 5*D*).

#### Step 5. Closure

The interval between the anterior and middle head of the deltoid is closed with No. 1 Vicryl (Ethicon, Inc.). Performing a partial osteotomy of the tip of the acromion allows for direct bone-to-bone healing.

Close in layers the subcutaneous plane and then the skin with Steri-Strips 3M.

### Post-operative rehabilitation

**Figure 6:**
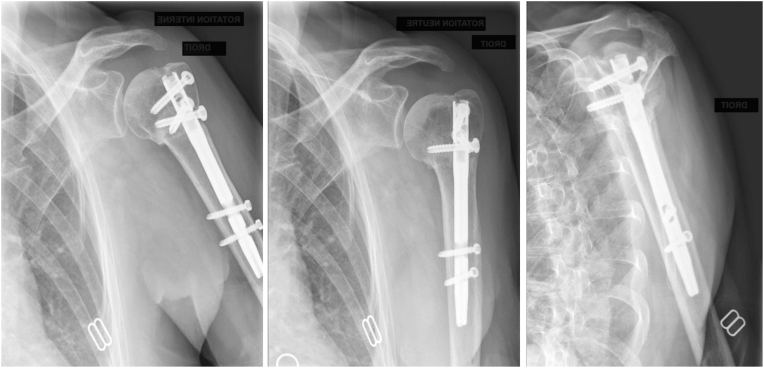
Post-operative anteroposterior and lateral radiograph.

Patients are discharged the day after surgery. The shoulder is placed in a neutral rotation sling for 3 weeks after surgery. Since day 1, the patient is encouraged to remove the sling for short periods 2-3 times per day to perform elbow flexion-extension and to perform shoulder pendulum exercises. After 3 weeks, rehabilitation with a physiotherapist is started with assisted active elevation and external rotation. Post-operative radiographs are routinely performed at follow-up (Fig. 6).

## Discussion

Despite being a common orthopedic injury, the optimal fixation method of 4-part PHFs remains a source of ongoing controversy, with relatively limited studies concentrating selectively on 4-part PHF.^1^^,^^8^^,^^11^^,^^15^^,^^25^

### IMN vs. locking plate for 4-part fractures?

Options included mainly OR and internal fixation with LPs, as IMN was used only for surgical neck fractures. However, due to improvements in nail and screw designs (from a curved to straight nail, respectively) and a greater understanding of surgical technique, indications have been extended also to 3- and 4-part fractures.^1^^,^^9^^,^^15^^,^^18^

Early IMNs had a proximal curvilinear design to enable a lateral entry point insertion avoiding to injury the humeral head cartilage.^2^^,^^13^^,^^19^ However, this approach has been associated to a greater risk of iatrogenic cuff tears and GT fractures during nail placement. Furthermore, in case of tuberosity fractures, the lateral entry position is frequently located at the level of the fracture zone, making the fixation challenging.

Straight nails need a more medial entry point, which increased the surrounding bone stock and implant stability.^13^

Biomechanically, the straight IMNs have 2 great advantages compared to LPs, helping to counteract displacing forces:

-a shorter lever arm due to transmission of the load medially, as highlighted extensively in femoral fractures.^20^^,^^24^
- another fixation point placed just below the humeral head subcortical bone, with the proximal nail tip working as an anchoring point.^6^

Clavert et al showed in a sawbone study that proximal humerus anterograde nails demonstrated increased stiffness and load to failure when compared to LPs for 4-part fractures.^4^

Further theoretical advantages of IMNs are less soft tissue dissection/disruption and preservation of blood supply (even in ORIN 4-part fractures, the distal approach for diaphyseal screws is avoided), a decreased risk of axillary nerve lesion at the level of the deep deltoid fascia, a decreased number of screws in the humeral head affecting the healing of the fracture and perfusion of the humeral head, a shorter operative time, a multiple plane of fixation, and a decreased direct cost (due to the number of locking screws used)^3^ (Table II).

**Table II:** Advantages.

1. IMN
Decreased number of screws in the humeral head
Multiple plane of fixation (antirotational)
Proximal nail tip fixation point
Shorten lever arm
Less soft tissue dissection/disruption and preservation of blood supply
Decreased risk of axillary nerve lesion at the level of the deep deltoid fascia
Shorter operative time
Reduced direct cost
Decreased complication and reoperation/revision rate
2. The superior approach
Parallel to Langer's lines (better cosmetic results)
Lowered risk of axillary nerve damage
Good visualization of the GT
The acromial osteotomy: direct bone-to-bone healing

*IMN*, intramedullary nailing

When compared to LPs, IMN has been reported to provide similar clinical outcomes but less complication and revision rate in treating 4-part PHFs.^3^^,^^4^^,^^8^^,^^21^^,^^23^

Shi et al in their metanalysis showed, without evaluating the results based on the type of fracture (2-, 3-, or 4-part fracture), that IMN is superior to LP in the treatment of PHFs in reducing the intraoperative complications, operative time, fracture healing, and risk of humeral AVN.^21^

Gadea et al also highlighted a difference in revision rate: 18% and 30%, respectively, for IMN and LPs.^8^ Interestingly, the need for implant (nail/plate) removal due to pain or stiffness but without any clear documented complication (eg, articular screw penetration, screw migration) was 1.8% for IMN and 17% for plates.

Differently, Wikerøy et al comparing a third-generation nail with plates for displaced 3- and 4-part PHFs, showed no statistically significant differences in function but surprisingly a higher complication (eg infection and AVN) and reoperation rate in the nail group at 2 years.^25^

Furthermore, the comparison between IMNs and LPs might not be interpreted correctly, having frequent demographic differences between patient populations (age and sex) and mixing different generation nails within the same group.^8^

Gadea et al in a multicenter study, compared LPs with IMN (both straight and curved) as a treatment for 4-part fractures.^8^ The clinical outcomes were significantly better in the plate group. However, the authors stated that 67% of patients having the curved nail had poor outcomes, whereas only 33 to 44% patients having a straight nail had poor outcomes.

### OR associated to the IN. Why?

Three radiological predictive factors increasing the risk of AVN and poorer functional outcomes have been identified: pre-operative calcar comminution, varus head reduction, and poor tuberosity reduction.^8^

The GT and LT contribute to vascularization of the humeral head and are the site of insertion of the anterior and posterosuperior rotator cuff. The correct tuberosity reduction is therefore mandatory to lower the risk of AVN and muscle atrophy. In case of a still inserted and functioning rotator cuff, the GT and LT are medialized and displaced, respectively, posterosuperiorly and anteriorly.

Therefore, unacceptable excessively displaced GT and LT should be corrected and reduced with an open approach: OR.

The superior or saber-cut approach is preferred to the more commonly anterolateral as it is parallel to Langer's lines, reaching a more cosmetic result and as it prevents to go too far down through the deltoid, therefore decreasing the risk of axillary nerve damage (Table II).

This approach enables a good visualization of the glenoid and GT, facilitating the reduction and does not violate any of the fracture fragments blood supply if compared to the anterior deltopectoral approach.

The partial osteotomy of the tip of the acromion is used to mobilizing the anterior deltoid head, while maintaining the strong tendon-to-bone attachment of the deltoid tendon insertion. The acromial osteotomy, allows a better direct bone-to-bone healing of the small fragment to the anterolateral aspect of the acromion, with a theoretically lower rate of nonunion.

A conventional deltopectoral approach has been proposed to be used for IMN.^16^ However compared to a deltoid split, leads to an easier access to the anterior structures of the proximal humerus but it is not useful in exposing also the posterior structures.^16^

There are limited studies that specifically evaluate the results of 4-part PHFs treated with IMNs and none of these evaluates the clinical results using the described ORIN approach for PHFs.^5^^,^^8^^,^^11^^,^^14^^,^^15^^,^^17^^,^^26^

When compared to 2- and 3-part, 4-part PHFs treated with IMN have been shown to be associated with lower clinical outcomes.^11^^,^^12^^,^^26^

Kloub et al evaluated 38 4-part PHFs at an average follow up of 57 months treated with a straight IMN through an anterolateral deltoid split approach.^14^ The authors observed 10 cases of complete humeral head necrosis (26.3%) and highlighted that reduction quality strongly influenced functional results and development of AVN. The incidence of AVN was 4.7% and 75%, respectively, in excellent and poor reduced 4-part fracture.

The same group evaluated 35 patients with a displaced 4-part PHF, treated with a different straight IMN through an anterolateral approach. The authors highlighted an AVN rate of 17%.^15^

Greenberg et al evaluated at a minimum of 1 year follow-up the clinical outcomes of 37 patients with a 4-part PHF treated with a straight IMN through a supralateral approach.^11^ No patient required hardware removal, and 5% of patients developed radiographic signs of AVN, but only 1,7% was symptomatic with shoulder functional limitation and pain.

Cuny et al observed the outcomes of a straight IMN for 4-part PHFs performed whether with a percutaneous access (in front of the acromion) or with an anterolateral approach.^5^ Surprisingly, the authors highlighted different results and complication rates (67 and 0%, respectively) whether the head fragment was valgus disengaged or impacted, supporting the idea that these 2 fractures represent different entities. The authors therefore suggested the use of IMN only for 4-part valgus-impacted joint fracture.

## Conclusion

We present a technique for the ORIN of 4-part PHFs.

## Disclaimers:

Funding: No funding was disclosed by the authors.

Conflicts of interest: Marc-Olivier Gauci is a consultant for Stryker and NewclipTechnics.Any additional authors, their immediate families, and any research foundations with which they are affiliated have not received any financial payments or other benefits from any commercial entity related to the subject of this article.
